# Navigating multiple pandemics: A critical analysis of the impact of COVID-19 policy responses on gender-based violence services

**DOI:** 10.1177/02610183221088461

**Published:** 2023-02

**Authors:** Tara Mantler, C. Nadine Wathen, Caitlin Burd, Jennifer C. D. MacGregor, Isobel McLean, Jill Veenendaal

**Affiliations:** 6221 Western University, Canada; 6221 Western University, Canada; 6221 Western University, Canada; 6221 Western University, Canada; University of British Columbia, Canada; 6221 Western University, Canada

**Keywords:** COVID-19, gender-based violence, interpretive description, multiple pandemics

## Abstract

COVID-19 illustrated what governments can do to mobilise against a global threat. Despite the strong governmental response to COVID-19 in Canada, another ‘pandemic’, gender-based violence (GBV), has been causing grave harm with generally insufficient policy responses. Using interpretive description methodology, 26 interviews were conducted with shelter staff and 5 focus groups with 24 executive directors (EDs) from GBV service organizations in Ontario, Canada. Five main themes were identified and explored, namely that: (1) there are in fact four pandemics at play; (2) the interplay of pandemics amplified existing systemic weaknesses; (3) the key role of informal partnerships and community support, (4) temporary changes in patterns of funding allocation; and (5) exhaustion as a consequence of addressing multiple and concurrent pandemics. Implications and recommendations for researchers, policy makers, and the GBV sector are discussed.

A pandemic can be understood as a condition that impacts people around the world (Grennan, 2019). When COVID-19 was declared a pandemic in early 2020 (World Health Organization, 2020), it became clear that governments would need to impose rigid public health restrictions to prevent the spread of this highly contagious disease. In Canada, as elsewhere, these restrictions included the use of masks/personal protective equipment, physical distancing, and stay-at-home orders (Cardenas et al., 2020; Crasta et al., 2020; Mohler et al., 2020). While these restrictions were necessary to reduce the transmission of COVID-19, there were significant unintended consequences for vulnerable populations, including those experiencing gender-based violence (GBV; Slakoff et al., 2020; van Gelder et al., 2020; Viveiros and Bonomi, 2020), those who are racialised (Addo, 2020; Leitch et al., 2020), and people using substances, especially street-procured opioids (Centers for Disease Control and Prevention, 2020; John Hopkins Bloomberg School of Public Health, n.d.). One consequence of COVID-19 being classified as a pandemic was the shift in public narratives used to describe GBV, i.e., that COVID-19 was a ‘pandemic,’ while GBV was a ‘shadow’ pandemic (Walters, 2020). However, the GBV pandemic is not new, having been deemed a major public health problem for decades (World Health Organization, 2021b).

GBV can be understood as any form of sexual, physical, mental, or economic harm perpetrated because of a person's gender. Physical and sexual forms of GBV within and outside intimate relationships impact an estimated one in three women globally (World Health Organization, 2021a), but this does not include other forms of gender-based harassment and exploitation, making actual prevalence rates much higher, especially among certain groups (World Health Organization, 2021a). The risk of experiencing GBV increases during crises (United Nations, 2021), with the COVID-19 pandemic being no exception. Canada's Assaulted Women's Helpline, and Canadian police services reported about double the number of calls related to domestic violence during the first six months of the COVID-19 pandemic compared to the same time period in 2019 (Thompson, 2021). Both hotline and police service call measures are imperfect, and likely underestimate actual incidents of GBV, as using both services requires access to a safe telephone; however, these proxies highlight the alarming increase in GBV.

In addition to these concerning trends, emerging research has shown how COVID-19, and policy and practice responses to it, have impacted women's health and safety, barriers to accessing services, as well as GBV services’ ability to respond to women's needs. Perpetrators have weaponised public health regulations such as ‘stay-at-home’ orders to increase their coercive control (Pfitzner et al., 2020) via increased surveillance and/or no longer allowing women to work outside the home, thus jeopardizing both the health and safety of women experiencing abuse as well as their ability to access services. There have also been major disruptions in GBV services as a result of COVID-19; specifically, maximum shelter occupancy has decreased substantially to adhere to COVID-19 policies for congregate settings, and there have been reported staffing challenges, as shelter workers were no longer able to work at multiple shelters (Ending Violence Association of Canada and Anova, 2020; Lyons and Brewer, 2021). Together, the increased health and safety concerns for women, barriers to accessing service, and disruptions to GBV services as a result of COVID-19 related policies and practices has further marginalised abused women during the COVID-19 pandemic.

We therefore argue that public health measures in response to the COVID-19 pandemic have had significant unintended consequences for the ongoing GBV pandemic, revealing and exacerbating longstanding issues regarding the vulnerability of both abused women and their children, as well as the organizations that serve them. Of note, we understand GBV has 'root causes' grounded in inter-related forms of structural violence. GBV is an inherently traumatic experience, while also having causal links with other forms of trauma and violence across the lifespan (Shonkoff et al., 2012). For example, GBV perpetration and victimization among adults is often rooted both in patriarchal gender norms, and in childhood experiences of violence in the home and the community (Courtois, 2008), and these traumatic experiences, and their consequences, accumulate (Scott-Storey, 2011). Given the high prevalence of trauma among Canadian adults, with about 76% experiencing at least one traumatic event meeting clinical thresholds in their lifetime (Van Ameringen et al., 2008; van der Kolk, 2015), it is little wonder that society's response to the COVID-19 pandemic has made GBV and its impacts worse.

The purpose of this study was to explore the effects of the COVID-19 pandemic, and policy responses to it, on the provision of GBV services, as described by staff and executive directors (EDs), in GBV organizations in Ontario, Canada.

## Methods design

This qualitative interpretive description study (Thorne, 2016; Thorne et al., 2004) used an integrated knowledge mobilization (KMb) approach (Kothari and Wathen, 2013, 2017). Interpretive description is aligned with a pragmatic (Morgan, 2014) orientation aiming to generate knowledge that is relevant in the context of applied disciplines (Thorne et al., 2004). This is very consistent with our integrated KMb approach, i.e., partnering with community organizations and leaders to collaboratively engage in mutually beneficial research. Below we provide an overview of the methods specific to data collected for the present analysis. Additional methods detail is available in Mantler et al. (2021).

## Sampling and recruitment

Purposive and snowball sampling were used, with our five partner EDs supporting recruitment of staff and their ED colleagues in selected regions in Ontario, supplemented by invitations for direct service staff to participate in the research sent via major GBV sector email list-servs in Ontario. Interested staff were asked to email the research team; individual interviews were conducted with shelter staff (n = 26) and five focus groups of four to six participants were completed with EDs (n = 24). Participants from this study were from 24 different agencies across Ontario, in both urban and rural areas.

## Participants

Staff and EDs included in this study served communities ranging from 4,700 to 1,500,000 people. Ten EDs and eight staff worked at rural GBV service organizations. Two EDs were from Indigenous organizations. Staff had worked for their various agencies ranging from less than a year to 30 plus years with the majority of staff in full-time positions (65%). The majority of EDs had a bachelor's degree and were an average of 48 years old (SD = 9.53).

## Procedures

Ethics approval was obtained from Western University's Non-Medical Research Ethics Board (Protocol 115865) and data collection occurred between June and October 2020. Participation for shelter staff consisted of a single video/telephone-based interview of approximately 60 min, and for EDs consisted of one two-hour focus group conducted by videoconference. Table 1 presents the questions asked during the interviews and focus groups. No participant in either the interview or focus group declined to answer or skipped any questions. All participants completed basic demographic questions.

**Table 1.** Questions asked during interviews and focus groups with shelter staff, and EDs.

Cohort	Questions
Interview with shelter staff	Tell me about the last few months – how have things been at the agency? What changed for you the most in your everyday work practice as a result of COVID-19? How was the timeline from when the pandemic started, to now? If you were giving your ED, or other shelters, advice right now about what changes to keep and what to get rid of, what would you say? How have these changes impacted your clients? Are there new stresses in your work, due to COVID-19 or other factors, that make it harder to care for your clients or yourself?
Focus group with EDs	How are things going for you in your shelter/service? What did you learn very quickly when COVID-19 restrictions emerged? What are the decision processes now? What is unique about values-based work? What have the big changes been? What lessons have you learned from COVID-19?

All interviews and focus groups were audio-recorded and transcribed verbatim by a professional transcription service. Each transcript was anonymised prior to analysis. The data collection and analysis process were guided by Lincoln and Guba’s (1985) and Thorne et al.’s (1997) principles of auditability, fit, dependence, and transferability. To reduce barriers to participation, shelter staff received a $50 gift card in recognition of their time. EDs were not provided an honorarium.

## Data analysis

Transcripts from both interviews and focus groups were organised using Quirkos qualitative analysis software (Quirkos, 2020). Interpretive description following Thorne's approach guided analysis (Thorne, 2016; Thorne et al., 2004). The 31 transcripts were each independently coded by two of the seven researchers involved. Initially, those who conducted the interviews/focus groups and the principal investigator who has extensive knowledge in the field, met and created a preliminary coding structure with definitions based on field notes, and what was known from the literature that had guided the interview questions. Each coding dyad was initially assigned two transcripts to analyse using open and line-by-line coding. The dyads met to discuss the applicability of the coding structure and code definitions, with refinements to the coding structure and definitions made, as needed. This process was repeated three times until the coding team was confident that the coding structure sufficiently covered the data, and was informed by extant practice literature, a fundamental principle of interpretive description. Next, all interview and focus group transcripts were assigned to two people for analysis. Once all transcripts were analysed, Quirkos files were merged across coders and queries were run to provide reports on each code and associated data related, for the present analysis, to the concept of ‘multiple pandemics.’ The coding team then met to theorise the relationship and structure of the data and extract meaning from it, an approach consistent with interpretive description (Thorne et al., 2004). Findings were then member-checked with the research partners during two half-day sessions, and recontextualised in the broader literature by the academic team (Thorne et al., 2004). For additional analysis details, see Mantler et al., 2021.

## Results

Our analysis illuminated five distinct but inter-related themes including: (1) there are in fact four pandemics at play; (2) the interplay of pandemics amplified existing systemic weaknesses; (3) the key role of informal partnerships and community support, (4) temporary changes in patterns of funding allocation; and (5) exhaustion as a consequence of addressing multiple and concurrent pandemics.

## Multiple pandemics at play

EDs and staff identified the emergence of pandemics beyond GBV and COVID-19, with one ED stating,

I’ve been navigating three pandemics, triple. There's COVID, absolutely, and then there's the issue of gender-based violence, and then anti-Black racism during this very critical time. Which has been extremely challenging and needing to really focus on how I care for myself, and how do I show up as a leader at a time where I need, a lot of support myself. Right? In order to just get through the day. Right? And deal with you know, look out for the needs of others. (FG5)

Moreover, these multiple pandemics are framed as inherently hierarchical, with the health implications of COVID-19 clearly, in the policies and guidelines being sent to services, outweighing all other concerns, including women's and children's safety, and efforts to counteract racism and provide a trauma- and violence-informed response to opioid use and overdose. These latter pandemics, as is the case historically, remained of lesser concern to governments and public health authorities. One ED described grappling with this realization and the impact it had on her, as follows,

Well, I mean, one of the pieces of cognitive dissonance that we’ve all had through this process is, holy crap, we’ve shut down society on a dime and have thrown everything we have at COVID, yet we’ve had our folks dying of overdose for years and there's been no resource, nothing that can be done. (FG3)

The fact that there was a swift and national public health response to the COVID-19 pandemic made evident this hierarchy. However, the public health response and restrictions that prioritised stopping the spread of COVID-19 also had very real ramifications on the GBV sector. Shelter staff and EDs struggled with the privileging of COVID-19 requirements and the associated changes in service provision, which were often in conflict with what was needed to address GBV and other factors affecting their clients. One staff member reflected on the intersection of COVID-19 and racism, explaining that she needed to lobby to allow women to participate in a Black Lives Matter march given the COVID-19 restrictions in shelter,

One of my personal, like, victories during Covid was I got approval to take a bunch of the women and their children to the Black Lives Matter march. … I fought pretty hard for it, just because we had a house of women who were all people of colour at the time. And it was something that was very important to me that these women be able to go. (S18)

The presence of multiple pandemics meant that shelter staff and EDs were contending with the impact of various requirements, which often clashed, on service processes and outcomes. The reality of the interaction of GBV, racism, and opioid use is not new; however, in a time where COVID-19 physical safety requirements were at the forefront of service delivery, this meant that women's needs, in terms of their abuse experiences and any substance use or racism-related issues, were not prioritised. Another staff member, for example, explained that Black women using shelter were often subjected to stricter guidelines around physical safety as they typically did not feel safe advocating for themselves or for exemptions to the rules the same way White women did. This subtle example illustrates how systemic racism permeates the culture of what have historically been, in Canada's shelters, predominantly White spaces,

So, then women return to the shelter, and it was, like, … stricter guideline put in place for [Black and Indigenous] women. Or the guideline that was in place for women to follow, while White women were able to make – give rationale of why they might need to be out and doing different things and excuse, and they know the system, while Black women and Indigenous women in the shelter were either afraid or don't want to ask for those pieces, but then you watch them suffer. (S14)

## The interplay of pandemics amplified existing systemic & structural weaknesses

Simultaneously trying to manage multiple pandemics amplified existing system-induced organizational weaknesses in areas from finance to service provision. One ED highlighted the difficulty experienced by shelters before and after COVID-19 saying: 'In gender-based violence it feels like this is a pandemic … so it was bad before and [COVID-19] just kind of compounds things' (FG3). One longstanding system weakness in the GBV sector is inadequate and inflexible funding (Burnett et al., 2016; Macy et al., 2010). When chronic underfunding was coupled with COVID-19 and the service changes required to meet public health restrictions for congregate living, enormous stress was placed on GBV services, especially women's shelters. One ED explained, 'Covid amplified everything that already was. So, where we had weaknesses, I would say in our finance department, damn they got bigger' (FG1).

Another longstanding issue in the GBV sector is a lack of housing to support women transitioning out of emergency shelter, i.e., 'second-stage' housing (Baker et al., 2010; Burnett et al., 2016; Raphael, 2001). When COVID-19 swept the world, many services were shuttered. One that remained closed for an extended period of time in Ontario was municipal housing, resulting in significant delays for processing applications and moving women and families to safe and affordable accommodations. One shelter staff member said, 'the housing issue has been like a challenge for the last four years unfortunately. And now it's obviously gotten even worse during the pandemic' (S6). This was echoed by EDs with one explaining,

So, you’ve got a community that's screaming ‘she needs to get in.’ We’ve got public health saying ‘uh-uh, these are the rules.’ And you’ve got women out there desperate to get in. Right? It's a mess. Because there's no f**king housing and [of] all of the thing[s] that the pandemic did to us, it highlighted already the craters that exist in the system. We’ve known this all along and now the whole world knows. (FG5)

## Informal partnerships and community support

In the face of multiple pandemics and increased stressors from amplified systemic weaknesses, GBV service organizations pulled together to support one another by sharing information and resources. Across Ontario, EDs created informal support groups to help navigate the pandemics. One ED, speaking to her peers within the focus group, described these informal partnerships as instrumental in supporting her through the early stages of the COVID-19 pandemic,

And I just want to thank all of our partnering agencies, just – I must say that you have been our strength as well because we’re able to collaborate with you and there's just so many different tables out there that we’re able to participate in just so we can help each other through this challenging time. (FG3)

Informal partnerships helped EDs to navigate the multiple pandemics and the increased stressors they experienced both as leaders and individuals. Other groups of EDs took the partnership a step further and started sharing resource information with each other and had regional discussions, instead of individual meetings, with the funding ministry to improve transparency and service coverage in the area. One ED, in a rural setting, explained how the group pushed back against separate meetings and created a new normal in their area, saying, 'And it's been funny because historically you’d get the ministry kind of saying, "Oh no, I have to meet with you separately" and we don't see why. Like, we know each other and each other[‘s] budgets. So, this has just allowed a different level' (FG4). The power of informal partnerships afforded EDs the opportunity to build community during a time when people were experiencing extreme isolation and enabled them to work together to navigate the many challenges of providing service during multiple pandemics.

Beyond the informal partnerships leveraged during COVID-19, staff and EDs talked about an outpouring of support from their communities both in terms of recognition of the vital service that these organizations provide, and through increased donations. One staff member explained they received many donations because their shelter is 'well-established in the community and we get a lot of donations, goods and gifts and gift cards and clothing' (S3). Some EDs credited the increase in donations and success of fundraising efforts to the presence of multiple pandemics. As one explained, 'So our fundraising has also gone through the roof. There's generosity in community. I found there is the parallel of Covid and Black Lives Matter super interesting and the way that organised bodies, like community services [fly into] action' (FG1). The generosity of many (but not all) communities during the COVID-19 pandemic provided organizations with funds to be able to react to the ongoing service change requirements; however, while fundraising efforts can help to support organizations, having to rely on fundraising for operations places them in a very precarious position.

During the first two months of COVID-19, GBV service organizations continued to experience underfunding; however, patterns of increased funding were observed once governments mobilised. For many EDs the sudden availability of funding from the government was shocking. One said,

So, all of a sudden COVID impacts us all, so now all of a sudden there's money. There's a f**king tap of money. Right? And everybody's like, oh here's money for the shelters. Oh, don't spend a dime on this, don't spend a dime on it. Whatever you need, it's coming back to you, it's coming back to you … It created the case that funding isn't such a f**king big deal for shelters because there's all of a sudden more money than we’ve ever seen in terms of a sector. (FG5)

For EDs it wasn't merely the increase in funding resulting from COVID-19 but also the change in accountabilities surrounding funding. Reporting requirements for funding changed from rigid pre-COVID-19 requirements that interfered with getting the work done, to almost unfettered flexibility that allowed EDs to respond to intersecting crises as they emerged. One ED highlighted this change saying,

And I mean we joked about it, that we had this COVID credit card, but we were spending money to meet need that needed to be met with this concept that we’ll figure out what we’re going to do as we roll into the third and fourth quarter of our budget year, but we just need to do what needs to be done to support that population. So, resource decisions were very equity-based, and that required us to make some choices about who needed our services the most. (FG3)

This change was reiterated by another ED who said,

I think that all of our money comes with the caveat in all funders … you can spend the money as you need to get through this time. Right? Whereas before if you wanted to change one f**king line, it was like asking God for permission. Right? Now it's like, oh yeah, no, do what you need to do to get through. (FG5)

The flexibility in funding allowed EDs to do the work that needed to be done; however, as the pandemic progressed, EDs started to notice the funding model reverting back to the previous more rigid approach. One explained, 'Yeah, at the beginning it was very much we were getting "the beans don't need to be counted anymore," and now we’re starting to hear "yeah, we’re going to start counting the beans again." It's a jarring return to reality' (FG3).

The re-implementation of rigid budgets was concerning for EDs, particularly when coupled with the fear of returning to a chronically underfunded and tenuous state. One said,

So, for me, COVID is part, is part of the same thing, right? Like we’re always, I feel we’re always responding to the pressures and the challenges and the social inequities in this sector. It's not new. But you know, in the sense of whether we have the resources to do that, that's always been a challenge. And I think that as we talked about, you know, in some respects the government stepped up and gave us some resources to do that. But then what? Because we were, we were doing this as if it was going to end, right? So, and so now then what? Right? Because we can only, the security alarm can only ring for so long before it dies, right? The battery runs out. (FG5)

Unfortunately, the tension between flexibility and rigidity of funding and the impact on the ability to do the work of shelters is not new. This tension, cyclical in nature, moves from rigidity where funders require dollars be spent in strict accordance with budgets, to flexibility, where EDs are able to set budgets but then reallocate money to be responsive to the emerging needs of individuals who experience GBV. One ED said, 'We have this macro management system that seems to work really well for us and not so well for our funders. And then it goes back to micromanagement where it works really well for our funders and it really f**king sucks for us' (FG1).

## Exhaustion as a consequence of addressing multiple and concurrent pandemics

Both EDs and staff found dealing with multiple pandemics was exhausting. This exhaustion stemmed from the daily changes to organizational policy, the lack of ‘normal’, and the reality that COVID-19 was one more pandemic to layer on to at least three others. The challenge of dealing with multiple pandemics meant that shelter staff and EDs were constantly changing policy and practice with no definitive end in sight. These fast-paced adjustments in the early stages of the pandemic were difficult for staff and EDs. One staff member said,

Since March, there have been so many changes in our procedures that I think it's sometimes hard for staff to keep up and remember sort of where we’re at and I think there can be a bit of exhaustion at some point when they’re like, OK one more change, then forget the last one and now we’re moving onto something different. I think that can be challenging. (S23)

This constantly changing environment left shelter staff and EDs yearning for a state of normalcy, which was not on the horizon when we spoke with them. One ED explained, 'It's been challenging. I think that we’re at the point right now where we’re all just sort of tired of dancing as fast as we can, as are the women. They want some sort of normalcy in their lives and are looking for us to provide that, and so that's not necessarily forthcoming from us' (FG1). Not knowing how long the pandemic would last or when things would get back to normal compounded feelings of exhaustion. EDs explained that despite trying to deal with changes as they evolved during the pandemic, it seemed like there was another issue that always emerged; this led to burn out. One ED said 'And I probably was the first person in this group [of EDs] that said, "I’m done folks. I’m burnt and I really don't care anymore. And yeah, you all should have a day off too so, go ahead, take them all"' (FG4). Exhaustion and burnout are not unique to shelter staff and EDs; these are consequences of the COVID-19 pandemic that were a reality for most people around the world. What is unique for our respondents was that the exhaustion and burnout was compounded by the fact that they had already been living through the GBV, racism, and overdose pandemics long before the emergence of COVID-19 – tantamount to starting a marathon when another is already underway.

## Discussion

This study explored the effects of the COVID-19 pandemic, and policy responses to it, on GBV service organizations in Ontario, Canada. Five interrelated themes were discussed : (1) there are in fact multiple pandemics affecting GBV services, including the two we set out to examine, along with systemic racism and the opioid-related overdose crisis; (2) the interplay of pandemics amplified existing systemic weaknesses in the GBV sector and beyond; (3) informal partnerships and community support were required for services to function according to their values, and indeed these flourished, (4) there were notable, but perhaps temporary, changes in patterns of funding allocation among GBV organizations; and (5) exhaustion among staff and EDs was an important consequence of addressing multiple concurrent pandemics. These are discussed below.

One impact of the confluence of having to address multiple pandemics in an already-stressed service environment was the emergence of a pandemic hierarchy. What became clear in policy decision-making was that the COVID-19 pandemic was the main priority, which resulted in new or revised discursive framings of the various other pandemics: GBV was a ‘shadow’ pandemic, anti-racism a ‘movement,’ and opioid-related overdoses a ‘crisis’ (Gomes et al., 2021; Leitch et al., 2020; Norton and Kerr, 2020; Walters, 2020; Webster et al., 2020). Situating these pandemics in a hierarchy creates the illusion that these pandemics are distinct and should be dealt with individually, when in reality they intersect in time, have overlapping root causes and people may have lived experience of more than one at a time. For example, we know that many people who experience GBV also experience racism (as well as other forms of discrimination and social exclusion) and use substances to cope with the consequences of their trauma experiences (Dlamini, 2021; Goldenberg, 2020; Sullivan et al., 2021). The GBV, racism and opioid-related overdose pandemics are longstanding, with mounting evidence that they worsened at an accelerated rate during the COVID-19 pandemic (Crooks et al., 2021; Devakumar et al., 2020; Gomes et al., 2021; Norton and Kerr, 2020; United Nations, 2021; World Health Organization, 2021a). However, the response to these pandemics has been, and continues to be, underwhelming at best. In fact, it can be argued that not only are these longstanding pandemics generally ignored or minimized by policy actors during 'normal' times, but that policy decisions past and present have made them worse. For instance, there are policies that actively work against supporting solutions for the GBV, racism, and opioid-related overdose pandemics such as dual charging by police in domestic violence cases, the use of ‘parental alienation’ to support abusers’ rights in cases of GBV that are being considered by courts, the Indian Act in Canada (which is explicitly racist and even genocidal), as well as the War on Drugs and anti-harm reduction approaches in policies being implemented across Canada, including using the COVID-19 pandemic as an excuse to close supervised consumption facilities (Cavalieri and Riley, 2012; Lapierre and Côté, 2016; Miller, 2001). In fact, Khanlou et al. (2021) noted that 'the pandemic's disproportionate risks and impacts bring into light historic, systemic, and structural inequalities at the intersection of racial and ethnic minority status, occupation, and class' (2021: 6) and underscore that purely biomedical solutions to COVID-19 (i.e. vaccines and treatments) will likely fail. Given the longstanding and intersecting nature of these pandemics before (Fronteira et al., 2021; Khanlou et al., 2021), and now during the COVID-19 pandemic, the reality is that, while they are capable and have found ways to support clients, GBV organizations and their staff are stressed to the brink of collapse.

The layering of multiple pandemics – what have been termed 'syndemics' (e.g. Khanlou et al., 2021) – is not the only stressor that GBV service organizations are experiencing; these pandemics intersect with longstanding systemic and structural weaknesses such as lack of adequate operational funding, funding inflexibility, and lack of safe and affordable housing for GBV survivors. This precarity of funding among marginalised organizations in Canada has been well-established (Lavoie et al., 2018). The impact of the COVID-19 pandemic on funding contracts was that they were short term and no longer followed predictable patterns and cycles of availability. There is a need for longer-term and more stable funding for GBV service organizations, particularly during crises. As a result of this variability in funding, organizations were forced to over-rely on donations to maintain their operating budgets, which places them at financial risk. COVID-19 also led to changes in funding practices such that EDs and staff were free to use funds in ways that were responsive to the needs of their clients, which was in stark contrast to strictness of pre-pandemic funding and reporting guidelines. However, this positive change was starting to revert to the more restrictive and inflexible approach by the time we conducted our later focus groups and interviews.

The longstanding barrier to women's safety – the need for safe and affordable housing (Baker et al., 2010; Menard, 2001) – was also made worse during the pandemic. Delays in housing applications became the norm as housing services closed for extended periods of time, leading to even longer wait lists, and putting more stress on emergency shelter stay limits, which are usually approximately three weeks long. Existing housing programs need to operate during crisis situations to ensure people can access affordable housing and not create a domino effect in both the women's and homeless shelter systems. There is also a need for additional emergency housing options that meet the needs of individuals experiencing GBV, as emerging evidence suggests that short-term hotel use during the pandemic was antithetical to the feminist values that underpin GBV work (Mantler et al., 2021).

GBV organizations have been working in a state of constant crisis for years. Therefore, the fact that they were able to find ways to work supportively to get through the first eight months of the COVID-19 pandemic is a testament to staff and ED commitment to the work being done. Adding the COVID-19 pandemic, and the inequitable, 'one-size-fits-all', constantly changing public policy response to the existing pandemics has led to profound exhaustion in the GBV sector. Despite this, leaders and staff worked together to support one another and leverage their formal and informal partnerships. Fully utilizing coordinating committees (regional multi-agency tables that coordinate service responses and advocacy) or similar structures is important going forward to sustain this collaborative momentum. Moreover, rural GBV service organizations exist in lower-resourced environments and are well versed in navigating workarounds such as sharing resources and leveraging ongoing supportive networks for leaders (Mantler et al., 2018; Peek-Asa et al., 2011). While these strategies have allowed rural organizations to maintain service excellence during the pandemic, they involve additional effort (and stress) on the part of rural staff and EDs. The COVID-19 pandemic took a serious toll on GBV organizations; funding and policy adjustments are required to ensure their recovery and ongoing well-being.

## Limitations and directions for future research

Data for this study was collected in the first eight months of the COVID-19 pandemic period; due to the ever-changing nature of the pandemic response, including lockdown restrictions and policy guidelines, the results reflect only its initial stages. Moreover, data were anonymised at the transcription level and as such, we are unable to conduct analysis in relation to participant characteristics. Longitudinal data collection as the pandemic evolved would have provided a more fulsome picture of the ongoing experiences of GBV service organizations during all stages of the pandemic. Follow-up studies would help us understand the latter pandemic stages, and the post-pandemic recovery. In ongoing analyses, our team has interviewed policy actors, public health personnel and sector advocates to examine decision-making as the pandemic continued. This will shed further light on how difficult it has been for all actors to address evolving risks and service requirements in a rapidly changing environment. Comparisons with findings from other policy jurisdictions, in Canada and internationally, would provide a broad picture of the interconnectedness of pandemics, lived experiences, and service and policy decisions and their impacts.

## Conclusion

Our findings illustrate that, when looking at the response to the COVID-19 pandemic in the context of GBV services, the Ontario policy response has not been equitable, nor, for some sectors, effective. While we saw that governments can rapidly mobilise a response and funding when faced with a pandemic, they generally choose not to do so for GBV, systemic racism or opioid-related overdose, as these pandemics are deemed of lesser importance. Moreover, the unintended consequences of a response to one pandemic (in this case, COVID-19) can cause additional and significant harm to those in the throes of the GBV, and other inter-related, pandemics. GBV service organizations have been dealing with multiple pandemics since long before COVID-19, existing in a state of chronic crisis while advocating for their organizations, and for societal change; this meant they were near exhaustion well before March 2020.

GBV service organizations in Ontario played (and, at the time of writing, continue to play) a critical role during the COVID-19 pandemic, offering services to women and children at risk of harm and even death while other services shut their doors- despite the fact that it took the Ontario government almost six weeks to declare them an ‘essential service,’ along with grocery stores. Throughout their 40+ year history in Canada, GBV service organizations have been there for women and children experiencing domestic violence, and they were there, despite significant policy-imposed barriers, during the COVID-19 pandemic. The strategies they used to continue to serve women required significant ingenuity, targeted opportunism, and formal and informal community support. We now need from government policy-makers and funders an acknowledgement of this crucial work, and serious policy attention to the ongoing pandemics of GBV, racism, and opioid-related overdose before the next crisis hits.

## References

[bibr1-02610183221088461] Addo IY (2020) Double pandemic: Racial discrimination amid coronavirus disease 2019. Social Sciences & Humanities Open 2(1): 100074. Elsevier. 34173502 10.1016/j.ssaho.2020.100074 PMC7574839

[bibr2-02610183221088461] Baker CK Billhardt KA Warren J, et al. (2010) Domestic violence, housing instability, and homelessness: A review of housing policies and program practices for meeting the needs of survivors. Aggression and Violent Behaviour 15(6): 430–439.

[bibr3-02610183221088461] Burnett C Ford-Gilboe M Berman H, et al. (2016) The day-to-day reality of delivering shelter services to women exposed to intimate partner violence in the context of system and policy demands. Journal of Social Service Research 42(4): 516–532. Taylor & Francis.

[bibr4-02610183221088461] Cardenas MC Bustos SS Chakraborty R (2020) A ‘parallel pandemic’: The psychosocial burden of COVID-19 in children and adolescents. Acta Paediatrica 109(11): 2187–2188. John Wiley & Sons, Ltd. 32799388 10.1111/apa.15536 PMC7461397

[bibr5-02610183221088461] Cavalieri W Riley D (2012) Harm reduction in Canada: The many faces of regression. In: John Wiley and Sons (eds.) Harm Reduction in Substance Use and High-Risk Behaviour: International Policy and Practice. London, England: Blackwell Publishing Ltd, pp. 382–394.

[bibr6-02610183221088461] Centers for Disease Control and Prevention (2020) Overdose Deaths Accelerating During COVID-19. Available at: https://www.cdc.gov/media/releases/2020/p1218-overdose-deaths-covid-19.html (accessed 9 July 2021).

[bibr7-02610183221088461] Courtois CA (2008) Complex trauma, complex reactions: Assessment and treatment. Psychological Trauma: Theory, Research, Practice, and Policy S(1): 86–100. American Psychological Association (APA). 10.1037/tra0000515 31580137

[bibr8-02610183221088461] Crasta D Daks JS Rogge RD (2020) Modeling suicide risk among parents during the COVID-19 pandemic: Psychological inflexibility exacerbates the impact of COVID-19 stressors on interpersonal risk factors for suicide. Journal of Contextual Behavioral Science 18: 117–127. Elsevier. 32923357 10.1016/j.jcbs.2020.09.003 PMC7476891

[bibr9-02610183221088461] Crooks N Donenberg G Matthews A (2021) Ethics of research at the intersection of COVID-19 and black lives matter: A call to action. Journal of Medical Ethics 47: 205–207. 10.1136/medethics-2020-107054 33547181

[bibr10-02610183221088461] Devakumar D Shannon G Bhopal SS, et al. (2020) Racism and discrimination in COVID-19 responses. The Lancet 395(10231): 1194. Lancet Publishing Group. 10.1016/S0140-6736(20)30792-3 PMC7146645 32246915

[bibr11-02610183221088461] Dlamini NJ (2021) Gender-based violence, twin pandemic to COVID-19. Critical Sociology 47(4–5): 583–590. SAGE Publications Sage UK: London, England. 10.1177/0896920520975465 PMC7723732 38603014

[bibr12-02610183221088461] Ending Violence Association of Canada and Anova (2020) *Pandemic meets pandemic: Understanding the impacts of Covid-19 on gender-based violence services and survivors in Canada*. Ottawa & London, ON. Available at: http://www.anovafuture.org/wp-content/uploads/2020/08/Full-Report.pdf (accessed 17 November 2021).

[bibr13-02610183221088461] Fronteira I Sidat M Magalhães JP, et al. (2021) The SARS-CoV-2 pandemic: A syndemic perspective. One Health (Amsterdam, Netherlands) 12: 100228. Elsevier. 33614885 10.1016/j.onehlt.2021.100228 PMC7887445

[bibr14-02610183221088461] Goldenberg SM (2020) Addressing violence and overdose among women who use drugs – need for structural interventions. JAMA Network Open 3(10). American Medical Association. DOI: 10.1001/JAMANETWORKOPEN.2020.21066. PMC8478364 33044547

[bibr15-02610183221088461] Gomes T Kitchen SA Murray R (2021) Measuring the burden of opioid-related mortality in Ontario, Canada, during the COVID-19 pandemic. JAMA Network Open. American Medical Association. DOI: 10.1001/jamanetworkopen.2021.12865. PMC8155822 34037734

[bibr16-02610183221088461] Grennan D (2019) What is a pandemic? JAMA 321(9): 910–910. American Medical Association. 30835310 10.1001/jama.2019.0700

[bibr17-02610183221088461] John Hopkins Bloomberg School of Public Health (n.d.) Opioid Crisis. Available at: https://www.globalhealthnow.org/tags/opioid-crisis (accessed 9 July 2021).

[bibr18-02610183221088461] Khanlou N Vazquez LM Pashang S, et al. (2021) 2020 syndemic: Convergence of COVID-19, gender-based violence, and racism pandemics. Journal of Racial and Ethnic Health Disparities: 1–13. Springer Science and Business Media Deutschland GmbH. DOI: 10.1007/S40615-021-01146-W/TABLES/3. 34648144 PMC8515913

[bibr19-02610183221088461] Kothari A Wathen CN (2013) A critical second look at integrated knowledge translation. Health Policy 109: 189–191. 10.1016/j.healthpol.2012.11.004 23228520

[bibr20-02610183221088461] Kothari A Wathen CN (2017) Integrated knowledge translation: Digging deeper, moving forward. Journal of Epidemiology and Community Health 71: 619–623. 28298415 10.1136/jech-2016-208490

[bibr120-02610183221088461] Lavoie JG. Varcoe C Wathen CN Ford-Gilboe M Browne AJ. (2018). Sentinels of inequity: examining policy requirements for equity-oriented primary healthcare. BMC Health Services Research 18(1): 1–12. 30200952 10.1186/s12913-018-3501-3 PMC6131743

[bibr21-02610183221088461] Lapierre S Côté I (2016) Abused women and the threat of parental alienation: Shelter workers’ perspectives. Children and Youth Services Review 65: 120–126. Pergamon.

[bibr22-02610183221088461] Leitch S Corbin JH Boston-fisher N, et al. (2020) Black lives matter in health promotion: Moving from unspoken to outspoken. Health Promotion International: 1–10. DOI: 10.1093/heapro/daaa121. 33305322 PMC7953963

[bibr23-02610183221088461] Lincoln YS Guba EG (1985) Naturalistic Inquiry. Beverly Hills, Calif: Sage Publications.

[bibr24-02610183221088461] Lyons M Brewer G (2021) Experiences of intimate partner violence during lockdown and the COVID-19 pandemic. Journal of Family Violence: 1–9. Nature Publishing Group. DOI: 10.1007/S10896-021-00260-X. PMC7908951 33654343

[bibr25-02610183221088461] Macy RJ Giattina MC Parish SL, et al. (2010) Domestic violence and sexual assault services: Historical concerns and contemporary challenges. Journal of Interpersonal Violence 25(1): 3–32. SAGE Publications Sage CA: Los Angeles, CA. 19252065 10.1177/0886260508329128

[bibr26-02610183221088461] Mantler T Jackson KT Ford-Gilboe M (2018) The CENTRAL hub model: Strategies and innovations used by rural women’s shelters in Canada to strengthen service delivery and support women. Journal of Rural and Community Development 13(3): 115–132.

[bibr27-02610183221088461] Mantler T Veenendaal J Wathen CN (2021) Exploring the use of hotels as alternative housing by domestic violence shelters during COVID-19. International Journal on Homelessness 1(1): 32–49.

[bibr28-02610183221088461] Menard A (2001) Domestic violence and housing: Key policy and program challenges. Violence Against Women 7(6): 707–720. SAGE Publications Thousand Oaks.

[bibr29-02610183221088461] Miller SL (2001) The paradox of women arrested for domestic violence: criminal justice professionals and service providers respond. Violence Against Women 7(12): 1339–1376. SAGE PublicationsThousand Oaks.

[bibr30-02610183221088461] Mohler G Bertozzi AL Carter J, et al. (2020) Impact of social distancing during COVID-19 pandemic on crime in Los Angeles and Indianapolis. Journal of Criminal Justice 68. Pergamon. DOI: 10.1016/J.JCRIMJUS.2020.101692. PMC7252124 32501302

[bibr31-02610183221088461] Morgan DL (2014) Pragmatism as a Paradigm for Social Research: *http://dx.doi.org.proxy1.lib.uwo.ca/10.1177/1077800413513733* 20(8): 1045–1053. SAGE PublicationsSage CA: Los Angeles, CA. DOI: 10.1177/1077800413513733.

[bibr32-02610183221088461] Norton A Kerr T (2020) Applying the lessons of COVID-19 response to Canada’s worsening opioid epidemic. EClinicalMedicine 29. Elsevier. DOI: 10.1016/J.ECLINM.2020.100633. PMC7677043 33235987

[bibr33-02610183221088461] Peek-Asa C Wallis A Harland K, et al. (2011) Rural disparity in domestic violence prevalence and access to resources. Journal of Women’s Health 20(11): 1743–1749. 10.1089/jwh.2011.2891 PMC3216064 21919777

[bibr34-02610183221088461] Pfitzner N Fitz-Gibbon K True J (2020) Responding to the ‘Shadow Pandemic’: Practitioner Views on the Nature of and Responses to Violence against Women in Victoria, Australia during the COVID-19 Restrictions. Monash University. DOI: 10.26180/5ED9D5198497C.

[bibr35-02610183221088461] Quirkos (2020) Quirkos.

[bibr36-02610183221088461] Raphael J (2001) Public housing and domestic violence. Violence Against Women 7(6): 699–706.

[bibr37-02610183221088461] Scott-Storey K (2011) Cumulative abuse: Do things add up? An evaluation of the conceptualization, operationalization, and methodological approaches in the study of the phenomenon of cumulative abuse. Trauma, Violence, & Abuse 12(3): 135–150. SAGE Publications Sage CA: Los Angeles, CA. 10.1177/1524838011404253 21511684

[bibr38-02610183221088461] Shonkoff JP Garner AS Siegel BS, et al. (2012) The lifelong effects of early childhood adversity and toxic stress. Pediatrics 129(1). DOI: 10.1542/peds.2011-2663. 22201156

[bibr39-02610183221088461] Slakoff DC Aujla W Penzeymoog E (2020) The role of service providers, technology, and mass media when home isn’t safe for intimate partner violence victims: best practices and recommendations in the era of COVID-19 and beyond. Archives of Sexual Behavior 49: 2779–2788. 32844303 10.1007/s10508-020-01820-w PMC7447204

[bibr40-02610183221088461] Sullivan C Block K Vaughan C (2021) The continuum of gender-based violence across the refugee experience. In: Cham (ed.) Understanding Gender-Based Violence: An Essential Tectbook for Nurses, Healthcare Professionals and Social Workers. Springer International Publishing, pp.33–47. DOI: 10.1007/978-3-030-65006-3_3.

[bibr41-02610183221088461] Thompson N (2021) Reports of domestic, intimate partner violence continue to rise during pandemic. Available at: https://www.cbc.ca/news/canada/toronto/domestic-intimate-partner-violence-up-in-pandemic-1.5914344 (accessed 9 July 2021).

[bibr42-02610183221088461] Thorne S (2016) Interpretive Description: Qualitative Research for Applied Practice. 2nd ed. Developing Qualitative Inquiry. London: Routledge. DOI: 10.4324/9781315545196.

[bibr43-02610183221088461] Thorne S Kirkham SR Macdonald-Emes J (1997) Interpretive description: A noncategorical qualitative alternative for developing nursing knowledge. Research in Nursing & Health 20(2): 169–177. 9100747 10.1002/(sici)1098-240x(199704)20:2<169::aid-nur9>3.0.co;2-i

[bibr44-02610183221088461] Thorne S Kirkham SR O’Flynn-Magee K (2004) The analytic challenge in interpretive description. International Journal of Qualitative Methods 3(1): 1–11.

[bibr45-02610183221088461] United Nations (2021) High Commissioner for Refugees- Gender-based violence. Available at: https://www.unhcr.org/gender-based-violence.html (accessed 9 July 2021).

[bibr46-02610183221088461] Van Ameringen M Mancini C Patterson B, et al. (2008) Post-traumatic stress disorder in Canada. CNS Neuroscience & Therapeutics 14(3): 171–181. CNS Neurosci Ther. 18801110 10.1111/j.1755-5949.2008.00049.x PMC6494052

[bibr47-02610183221088461] van der Kolk B (2015) The Body Keeps the Score: Brain, Mind, and Body in the Healing of Trauma. New York, NY: Penguin.

[bibr48-02610183221088461] van Gelder N Peterman A Potts A, et al. (2020) COVID-19: Reducing the risk of infection might increase the risk of intimate partner violence. EClinicalMedicine 21. Lancet Publishing Group. DOI: 10.1016/J.ECLINM.2020.100348. PMC7151425 32292900

[bibr49-02610183221088461] Viveiros N Bonomi AE (2020) Novel coronavirus (COVID-19): Violence, reproductive rights and related health risks for women, opportunities for practice innovation. Journal of Family Violence: 1–5. Springer. DOI: 10.1007/S10896-020-00169-X. PMC7275128 32836735

[bibr50-02610183221088461] Walters J (2020) COVID-19 shelter-at-home orders: Impacts and policy responses in the context of intimate partner violence. World Medical & Health Policy 12(4): 533–539. John Wiley & Sons, Ltd.

[bibr51-02610183221088461] Webster F Rice K Sud A (2020) A critical content analysis of media reporting on opioids: The social construction of an epidemic. Social Science & Medicine 244. Pergamon. DOI: 10.1016/J.SOCSCIMED.2019.112642. 31731136

[bibr52-02610183221088461] World Health Organization (2020) Rolling updates on coronavirus disease (COVID-19). Available at: https://www.who.int/emergencies/diseases/novel-coronavirus-2019/events-as-they-happen (accessed 9 July 2021).

[bibr53-02610183221088461] World Health Organization (2021a) Devastatingly pervasive: 1 in 3 women globally experience violence. Available at: https://www.who.int/news/item/09-03-2021-devastatingly-pervasive-1-in-3-women-globally-experience-violence (accessed 8 July 2021).

[bibr54-02610183221088461] World Health Organization (2021b) Violence against women. Available at: https://www.who.int/news-room/fact-sheets/detail/violence-against-women (accessed 17 November 2021).

